# Immune Components in Human Milk Are Associated with Early Infant Immunological Health Outcomes: A Prospective Three-Country Analysis

**DOI:** 10.3390/nu9060532

**Published:** 2017-05-24

**Authors:** Daniel Munblit, Marina Treneva, Diego G. Peroni, Silvia Colicino, Li Yan Chow, Shobana Dissanayeke, Alexander Pampura, Attilio L. Boner, Donna T. Geddes, Robert J. Boyle, John O. Warner

**Affiliations:** 1 Department of Paediatrics, Imperial College London, London W2 1NY, UK; lychow8@gmail.com (L.Y.C.); r.boyle@nhs.net (R.J.B.); j.o.warner@imperial.ac.uk (J.O.W.); 2 Faculty of Pediatrics, Federal State Autonomous Educational Institution of Higher Education I.M. Sechenov First Moscow State Medical University of the Ministry of Health of the Russian Federation., Moscow 119991, Russia; 3 International Inflammation (in-FLAME) network of the World Universities Network, Perth 6000, WA, Australia; 4 Allergy Department, Veltischev Clinical Pediatric Research Institute of Pirogov Russian National Research Medical University, Moscow 125412, Russia; trenevamarina@mail.ru (M.T.); apampura1@mail.ru (A.P.); 5 Department of Clinical and Experimental Medicine, Section of Paediatrics, University of Pisa, Pisa 56126, Italy; diego.peroni@unipi.it; 6 National Heart and Lung Institute, Imperial College London, London SW3 6NP, UK; s.colicino@imperial.ac.uk; 7 Royal Holloway University of London School of Biological Sciences, Biomedical Sciences, London TW20 0EX, UK; shobanadis@hotmail.com; 8 Department of Life and Reproduction Sciences, Section of Paediatrics, University of Verona, Verona 37124, Italy; attilio.boner@univr.it; 9 School of Molecular Sciences, The University of Western Australia, Perth, WA 6009, Australia; donna.geddes@uwa.edu.au; 10 National Institute of Health Research Collaboration for Leadership in Applied Health Research and Care for NW London, London SW10 9NH, UK

**Keywords:** colostrum, human milk, immune modulators, immunologically active molecules, cytokines, growth factors, health outcomes, immunological outcomes

## Abstract

The role of breastfeeding in improving allergy outcomes in early childhood is still unclear. Evidence suggests that immune mediators in human milk (HM) play a critical role in infant immune maturation as well as protection against atopy/allergy development. We investigated relationships between levels of immune mediators in colostrum and mature milk and infant outcomes in the first year of life. In a large prospective study of 398 pregnant/lactating women in the United Kingdom, Russia and Italy, colostrum and mature human milk (HM) samples were analysed for immune active molecules. Statistical analyses used models adjusting for the site of collection, colostrum collection time, parity and maternal atopic status. Preliminary univariate analysis showed detectable interleukin (IL) 2 and IL13 in HM to be associated with less eczema. This finding was further confirmed in multivariate analysis, with detectable HM IL13 showing protective effect OR 0.18 (95% CI 0.04–0.92). In contrast, a higher risk of eczema was associated with higher HM concentrations of transforming growth factor β (TGFβ) 2 OR 1.04 (95% CI 1.01–1.06) per ng/mL. Parental-reported food allergy was reported less often when IL13 was detectable in colostrum OR 0.10 (95% CI 0.01–0.83). HM hepatocyte growth factor (HGF) was protective for common cold incidence at 12 months OR 0.19 (95% CI 0.04–0.92) per ng/mL. Data from this study suggests that differences in the individual immune composition of HM may have an influence on early life infant health outcomes. Increased TGFβ2 levels in HM are associated with a higher incidence of reported eczema, with detectable IL13 in colostrum showing protective effects for food allergy and sensitization. HGF shows some protective effect on common cold incidence at one year of age. Future studies should be focused on maternal genotype, human milk microbiome and diet influence on human milk immune composition and both short- and long-term health outcomes in the infant.

## 1. Introduction

Increasing rates of allergy/atopy are of great concern with children bearing the greatest burden of this increase [1]. Long-term disease and health outcomes have been associated with multiple factors but, in particular, maturation of the immune system and immune-mediated diseases have been linked to later metabolic disorders such as cardiovascular disease [2]. Whilst there is evidence to suggest that the maternal environment in pregnancy has an impact on subsequent development of immune system in their offspring [3], early nutrition in the first two years of life, a rapid period of infant growth and development, is also believed to impact short- and long-term health. A plethora of evidence exists describing the multiple benefits of breastfeeding for both the mother and the infant, yet the mechanisms by which they confer these benefits remain to be fully elucidated.

Human milk (HM) contains a large variety of active immune components [4], which are present in differing concentrations [5], yet no comprehensive study has delved into the influences of maternal characteristics on immune composition of HM. We may expect HM to contain all necessary immunological components in the amounts needed for an appropriate infant immune development. Abundance of immune active molecules in HM represents a major difference when compared with any formula milk available. Further, little is known about the immune composition’s influence on short- and long-term health outcomes. 

It can be expected that HM composition may naturally change as a response to a number of environmental and genetic factors. HM immune components may influence immunological health outcomes but linear relationships between HM composition and subsequent health outcomes should be evaluated with care, due to potential confounding. Previously reported differences in HM immune composition between the countries and within the same populations provide evidence of volatility and reasons behind it remain unclear.

Amongst immunologically active molecules studied in HM, transforming growth factor β (TGFβ) is the most well described. HM contains all the isoforms of TGFβ, with TGFβ2 being the most dominant; accounting for 95% of the total TGFβ [6]. Protective effects of TGFβ on gut mucosal inflammation have been shown in animal models [7]. This protective effect may be explained by promotion of IgA production and induction of oral tolerance [8,9]. Oddy and Rosales systematically reviewed human milk TGFβ ability to influence infantile immunological outcomes [10]. Most of the studies included in the analysis found a statistically significant association between higher TGFβ concentration in HM and reduced risk of atopic diseases in the child. The authors suggested that TGFβ found in the milk may play a role in homeostasis maintenance in the intestine, regulating inflammation and subsequently promoting oral tolerance which may reduce the risk of allergy development.

Cytokines are one of the major classes of immune components in HM [11] but they are present in low concentrations; therefore, there is some doubt as to whether they have significant biological activity sufficient to modify health outcomes. On the other hand, growth factors are detected in much higher quantities in both colostrum and mature milk [12] and may therefore have a more potent influence in facilitating immunity maturation, as has been shown in both animal models [13] and human studies [14,15].

Hepatocyte growth factor (HGF) is a less-studied component that is also present in HM in high amounts, comparable with those of TGFβ [12,16,17], but has received less attention. HGF levels in human colostrum are 20 to 30 times higher when compared to paired maternal serum [16], suggesting that this growth factor is actively secreted into breast milk and may have an important role in early immune development. HGF is assumed to be in such high concentrations to facilitate proliferation, angiogenesis and intestinal tissue maturation via paracrine and endocrine signaling [12,17]. Accumulated knowledge from animal model studies suggests that HGF may also play a role in airway hyper-responsiveness and airway remodeling in allergic asthma [18,19,20] which highlights potential importance of this immune active molecule for human milk research.

We aimed to assess the impact of maternal and environmental factors in relation to colostrum and mature human milk immune active components composition and health outcomes at the age of six and twelve months. 

## 2. Materials and Methods 

### 2.1. Study Setting, Eligibility Criteria, and Ethics

The investigations and sample collection have been conducted following ethical approval by Ethics committees in three countries participating in the study: West London Rec 3 (UK) (Ref. number 10/H0706/32) and all paperwork has been completed according to the hospital R & D Joint Research Office (UK) (JROSM0072) policy; the Ethical Committee of the Azienda Ospedaliera di Verona (Italy) (approval No. 1288), and the Moscow Institute of Paediatrics and Child Health of the Ministry of Health of the Russian Federation (Russia) (approval No. 1-MS/11). All women provided written informed consent.

Women were enrolled at antenatal and postnatal units of 3 participating centers: Maternity Hospital No. 1, Moscow, Russia; St. Mary’s Hospital, London, UK; G.B. Rossi Hospital, Verona, Italy. Details of recruitment are described in full elsewhere [21].

### 2.2. Medical Records and Interview

Following enrolment, participants underwent allergy skin prick testing (SPT) and answered a 10-minute interview-based questionnaire regarding their medical history. Exposure variables recorded were selected based on a detailed review of known determinants of HM composition [22]. Information collected from the recruited women included: parity; age; mode of delivery; details of residence environment, such as mould presence at home, regular contact with animals and/or pets at home; exposure to tobacco smoke (smoker or living in household with smoker or self-reported passive smoker); any reports of infections during pregnancy. We also obtained information on maternal dietary preferences—fish, fresh fruit, and probiotic intake. Participant medical records were reviewed by study personnel to extract relevant health information which was not available from questionnaires, prior to breast milk analysis. SPT was undertaken using the following solutions: Histamine 1% Positive Control, Glycerol Negative Control, House Dust Mite (*Dermatophagoides pteronyssinus*), Cat (*Felix domesticus*), Grass Pollen, Birch pollen, Peanut, Hazelnut, Egg (all from Stallergenes, SA 92160 Anthony, France), and Cow’s milk (ALK-Abello, Hǿrsholm, Denmark). SPT was performed by standard technique using 1 mm lancets (ALK-Abello, Hǿrsholm, Denmark), and were read at 15 min. Allergic sensitization was defined as a wheal ≥3 mm to at least one allergen, in the context of a wheal ≥3 mm to histamine and no wheal to the negative control.

### 2.3. Human Milk Sampling 

Participants were given sterile tubes to collect their own colostrum (once in the first 6 days of life) and mature HM (once at 4–6 weeks postpartum). Instructions were given for collection of samples by manual expression or by collecting the drip from the contra-lateral breast during feeding [23]. Colostrum samples were frozen at −50 °C to −80 °C within 12 h of collection. HM samples were collected at home, transported to participating units by study staff, and frozen at −50 °C to −80 °C within 12 h of collection. It has been previously demonstrated that storage for 6 months at either −20 °C or −80 °C did not influence the concentration of immune active factors in human milk [24]. After thawing, samples were centrifuged at 1500× *g* for 15 min at 4 °C. The lipid layer was removed with a pipette and aqueous fraction was analysed for immune modulators [25]. All milk samples were transported to London at −70 °C where the samples were stored at −80 °C until analysis.

### 2.4. Electro-Chemiluminescence

We used electro-chemiluminescence to measure immune mediators in colostrum and breast milk samples for Th1 and Th2 cytokines, HGF, and TGFβ1-3 (MesoScale Discovery, Rockville, MD, USA). Laboratory experiments were run according to manufacturer’s protocol, using an eight-point standard curve. Assays were run in duplicate with no dilution used for Th1 and Th2 cytokines (IL2, IL4, IL5, IL10, IFNγ, IL12, IL13) and HGF, and 1:2 dilution for TGFβ assays, following pilot experiments which showed that TGFβ2 levels in undiluted milk samples were often greater than the upper limit of detection. For TGFβ analysis, samples were acidified by the addition of 1N HCl to colostrum or mature milk. Acidified samples were then neutralised by addition of 20 μL of 1.2 NaOH, 0.5 HEPES. Assays were run in duplicate, and mediator levels were excluded where the CV was >25%.

### 2.5. Health Outcomes

The infants’ health outcomes were assessed at the age of 6 months by means of a phone questionnaire and at one year of age by a questionnaire during the follow-up visit. All questions were carefully explained to the mothers. All health outcomes were self-reported by the mother except atopic sensitization which was assessed by skin prick test. Information on fever, wheeze, common cold, eczematous rash, reflux and vomiting cumulative incidence, food allergy/sensitivity or intolerance incidence were solicited.

Common cold was defined as at least one episode of runny nose or cold lasting for a minimum of 3 days. Cough/wheeze outcome was defined as at least one episode of recurrent cough or wheezing prior to assessment at one year of age. Eczema symptoms were considered present if the child had ever had a characteristic itchy eczematous rash intermittently at any time during the last 6 months. Parental-reported food allergy was defined as food allergy/sensitivity or intolerance incidence reported by the parents within the first 12 months of life. An infant was considered to be atopic if s/he had positive control wheal of ≥3 mm and any of the allergen-induced wheals being ≥3 mm greater than negative control.

For exclusive breastfeeding, the World Health Organisation (WHO) recommended definition “that the infant receives only breast milk” was used. No other liquids or solids are given—not even water—with the exception of oral rehydration solution, or drops/syrups of vitamins, minerals or medicines” [26].

### 2.6. Statistical Analysis

We assessed the probability of infants to develop particular health outcomes (presented as a binary variables) depending on potential determinant, using a binomial GLmulti (GLM) and LASSO (Least Absolute Shrinkage and Selection Operator) analyses. The resulting term importance was plotted in order to graphically visualize the effects of determinants. Such plots provide a graphical representation of importance estimate or relative evidence weight of candidate determinants. These weights are computed as the sum of the relative evidence weights of all possible models in which the term appears. Sensitivity analysis was performed for both methods and all models; a different number of variables were included to make sure that those selected had a real and credible effect on the outcome. 

Health outcomes assessed using models were: fever, eczema, food allergy/sensitivity/intolerance assessed at 6 months. Common cold incidence, reflux and/or vomiting, and cough/wheeze were assessed both at 6 and 12 months. Influence of immune active molecules level in colostrum and mature HM in conjunction with potential confounding factors was analysed in relation to health outcomes at the age of 6 and 12 months. The most reliable information was available for eczema, cough/wheeze and food allergy at 6 months and common cold at 12 months, these data were used as health outcomes for the statistical modelling. 

The following determinants of health outcomes were included into the models: levels of growth factors in colostrum and mature human milk, detectability of cytokines in colostrum and breast milk, site of collection, colostrum collection time, maternal atopic status, delivery type, infant gender. Prior to inclusion, descriptive statistics, cross-tables, correlation and descriptive statistical tests were performed on each determinant to describe the importance of each variable and identify which may be the most useful in explaining and predicting a particular health outcome. Human milk immune active molecules assessed were presented as levels of HGF, TGFβ1, TGFβ2, TGFβ3, and cytokines (IL2, IL4, IL5, IL10, IFNγ, IL12, IL13) detectability. As cytokines are detected in human milk in low concentrations, data were transformed into binary variable (detectable vs. undetectable) to increase statistical analysis power. Data on cytokine detectability can be found elsewhere [21]. 

Statistical analysis was performed using R statistical package version 3.1.0 (R Core Team. R: A language and environment for statistical computing. R Foundation for Statistical Computing, Vienna, Austria). As a first step, univariate analysis and correlation matrix were used, followed by multivariate analysis which included modelling, using LASSO and GLM.

## 3. Results

### 3.1. Study Population

A total of 481 mothers were recruited into the study from June 2011 to March 2012 from the birth centres and antenatal and postnatal units of secondary and tertiary hospitals from three countries, located in Northern Europe, Eastern Europe, and the Mediterranean area. Of 481 women, 398 (UK *n* = 101, Russia *n* = 221, Italy *n* = 76) provided samples and were included in this study. The 83 mothers unwilling or unable to provide colostrum samples were not evaluated further.

Demographic data of the participants is described in full elsewhere [21]. Data on co-variates included into multivariate statistical analysis and health outcomes is presented in Table 1. Significant differences between groups were seen for most variables recorded. Maternal allergic sensitization (highest in UK), tobacco smoke exposure (highest in Russia), rate of infant allergic sensitisation (highest in Italy), parental-reported food allergy and common cold (highest in Russia), and parental-reported cough and wheeze (highest in UK), all differed significantly. Rates of maternal atopy, parity and infant gender did not differ for health outcomes assessed (Table A1,Table A3,Table A5 and Table A7).

### 3.2. Associations between Determinants and Infant Health Outcomes

When unadjusted levels of growth factors and detectability of cytokines were assessed, we found that detectable IL13 and IL2 in breast milk were significantly less often associated with eczema at 6 months. In contrast, TGFβ2 in breast milk was associated with eczema, but this did not reach statistical significance. Lower levels of HGF were found in colostrum of the mothers of infants who subsequently developed common cold at 12 months of age. No other factors were found to be associated with any health outcome (Table A1, Table A2, Table A3, Table A4, Table A5, Table A6, Table A7 and Table A8).

**Figure 1:**
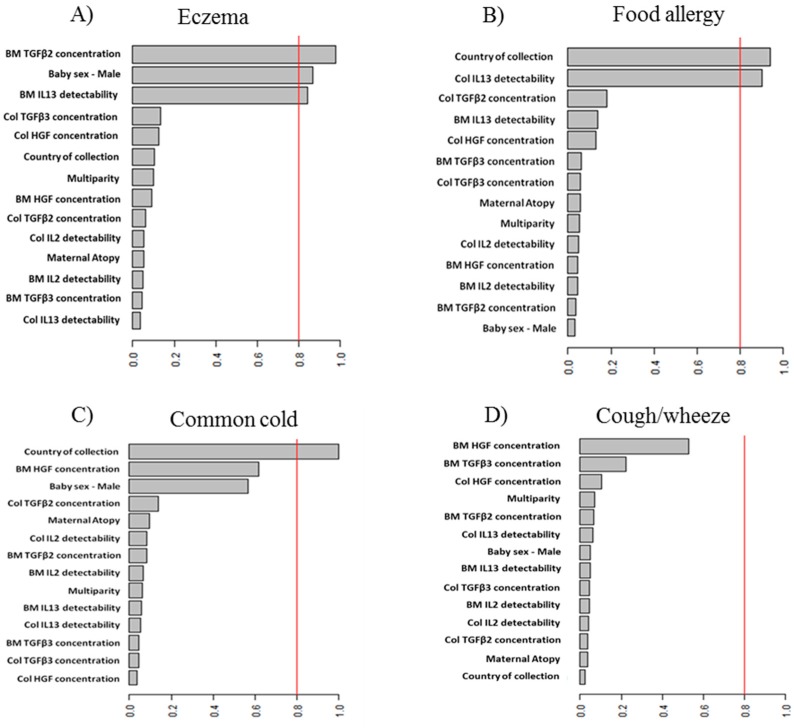
“Best” statistical model for each outcome: (**A**) eczema; (**B**) food allergy; (**C**) common cold; (**D**) cough/wheeze. Each model highlights those factors having the most significant influence on a particular outcome development, out of all determinants assessed. These are relative evidence weights of the covariates, with scale between 0 and 1.0 (equivalent of 0% to 100%). These weights are computed as the sum of the relative evidence weights of all models demonstrating presence of a particular determinant out of all models assessed in which the covariate appears. All results are adjusted. Col. stands for colostrum; BM-mature breast milk.

When multivariate analysis was performed, “Best” statistical model for each outcome highlighted those factors having the most significant influence on a particular outcome. All results are adjusted for the site of collection as a potential confounder. The average importance of the variables for a particular health outcome across all possible models visualised is shown in Figure 1. All factors found to be important at least in a single model and any significant associations with health outcomes are presented in Table 2.

#### 3.2.1. Associations between Maternal Factors/Environment and Health Outcomes

Maternal/environmental determinants reviewed were not found to be important for health outcomes development. At least one episode of common cold at the age of 12 months was reported less often OR 0.02 (95% CI 0.01–0.23) by mothers living in Moscow. Country of sample collection was not associated with parental-reported food allergy OR 0.13 (95% CI 0.01–1.46) and OR 2.07 (95% CI 0.47–9.02) for Verona and Moscow respectively. 

#### 3.2.2. Association between Collection Site and Colostrum/Breast Milk Composition

Among the studied variables, three were associated with eczema development at 6 months postpartum. Infants having breast milk with higher levels of TGFβ2 were at significantly higher risk OR 1.04 (95% CI 1.01–1.06) of eczema development. In contrast, if detectable levels of IL13 were present in HM, it was associated with a reduced risk OR 0.18 (95% CI 0.04–0.92) of eczema development. These results confirm the outcomes of the univariate analysis. In our cohort, boys tended to have eczema less often OR 0.2 (95% CI 0.05–0.84) compared with girls. Children fed colostrum with detectable levels of IL13 in colostrum were found to be less likely OR 0.1 (95% CI 0.01–0.83) associated with parental-reported food allergy at the age of 6 months. 

HGF concentration in breast milk was associated with less common cold development OR 0.19 (95% CI 0.04–0.92). No determinants were found to be associated with cough/wheeze development at the age of 12 months. There is some tendency for HGF to be associated with a higher risk of cough or wheeze development, but results did not reach significance OR 1.89 (95% CI 0.94–3.78). 

There was no association between any factor and infant allergic sensitisation at one year of age. It may be explained by a very small sample size as only 14 babies from the three cohorts developed allergic sensitisation.

## 4. Discussion

Mothers from the three geographical regions studied live in very different environments, have differences in diet, lifestyle and it is necessary to take into account many factors which may potentially influence human milk composition as well as health outcomes. In contrast to other studies, we included a large number of potential determinants, adjusting the results to the site of collection as a potential confounder making the results more robust. 

The aim of the study was to investigate whether peptide regulatory factors in human milk impacted infant health outcomes. Despite limitations, the results suggest that variability of human milk composition has an effect on parental-reported eczema, food allergy, and common cold development. The results support the concept that IL13 and HGF have protective effects and TGFβ2 may act as a risk factor, which may explain the diversity of results from epidemiological studies on the health-promoting effects of breastfeeding with regards to allergic diseases. Further, many studies attempted to evaluate colostrum and human milk composition influence on atopy and/or allergy development in the first years of life and produced conflicting results [23,27,28,29]. 

As TGFβ is an important regulatory cytokine suppressing both Th-1 and 2 activity, it appears counter-intuitive that high levels are associated with increased risk of eczema development. However, eczema is primarily a condition of impaired skin barrier function with allergic sensitization being a secondary phenomenon. Our findings suggest that higher concentrations of TGFβ2 in mature human milk are associated with significantly increased risk of eczema reported by the age of 12 months. Some studies support this observation, having found increased TGFβ1 and TGFβ2 in colostrum associated with the eczema onset in infants [6,28,29]. However, conflicting results have come from other studies [23,27,30,31,32]. It is not known why TGFβ might have an adverse effect on eczema, but it may be related to a particular isoform influence. A known classic example of TGFβ isoform differences has been observed in cutaneous scarring experiments done on animal models [33]. In these experiments mammalian embryos showed healing with no scarring and full skin recovery [34] with the expression of high levels of TGFβ3. In contrast, low levels of TGFβ1 and 2 [35] as well as similar TGFβ3 function have been demonstrated in human scarring physiology [36]. These pathways suppress immune surveillance in the skin such that infections break down the already genetically susceptible tissue. TGFβ family proteins are known for their diversity in biological functions, including migration of normal and abnormal cells, as shown in cancer research [37]. This may be a mechanism behind an association between the levels of TGFβ2 in HM and eczema. High concentrations of TGFβ2 may represent a biomarker that may predict risk of eczema in breastfed infants. This provides the initial inflammatory trigger to establish skin inflammation. Only a small number of children in our cohort were sensitized, which does not allow for an accurate assessment of a decreased barrier function; however, TGFβ may be impacting the skin barrier independent of allergy. However, the most likely explanation is that TGFβ in HM is a key factor, playing an important role in gut integrity maintenance as well as oral tolerance induction [38,39].

Detectable IL13 in mature milk reduced the risk of parent-reported eczematous rash development, whilst detectability of this cytokine in colostrum seems to have a similar association with food allergy/sensitivity/intolerance reported by the mothers at six months of age. This is the first study to report this association between IL13 in colostrum/human milk and eczema and/or food adverse events in children at 12 months of age. Eczema is not primarily an allergic condition but a heterogeneous disease. It may be speculated that a combination of TGFβ and IL13 may lead to TH1 responses impairment. The high IL-13 in colostrum fits well with the presumed impact on Immunoglobulin (Ig) E production but the counter effect of detectable levels in mature milk may be irrelevant as they are so low as to have little if any biological significance. Although maternal-reported events are a subjective measure and risk of bias is increased, these data should be validated on larger cohorts of high-risk infants. 

Most studies agree that levels of other human milk immune active molecules, cytokines in particular, are not associated with atopy and/or allergy development in early life [23,27,29,30]. Only 14 infants developed allergic sensitisation in our cohort, which therefore provided insufficient statistical power to analyse for any associations.

Human milk has been shown to be beneficial for infants in part by providing high concentrations of immune active molecules which are associated with a decreased incidence of neonatal respiratory infections as well as long-term outcomes, such as wheeze and/or asthma [40]. Gdalevich and Mimouni provided a meta-analysis suggesting protective effects of exclusive breastfeeding in the first months of life on later asthma development [41]. However, the difficulty in combining data is that the wheeze/asthma phenotype differs between studies. Further, it is known that most early life wheezers do not subsequently develop persistent asthma.

Our data did not show any significant association of colostrum and/or HM immune active molecules with parent-reported cough or wheeze episodes at 12 months of age. It is known that a large proportion of infants suffer asymptomatic infections during the first year of life [42]. There was also no association between human milk composition and common cold reported at 6 months of age. However, when assessed at one year of age, HGF levels in HM were associated with reduced incidence of common cold reported at 12 months of age. HGF is known for its ability to provide protection during inflammatory diseases, directly targeting macrophages or lymphocytes [20]. Animal research showed that HGF administration stimulates intestinal cell proliferation resulting in intestinal growth induction [43]. HGF may well act in a similar fashion when transferred in high amounts into infants’ gut. This finding shows that HGF as a component of human milk may not only play a very important role during the very first days of life, providing infant gut immunity development and maturation, but it is also capable of influencing long-term health outcomes. However, we cannot rule out a reversed causative relationship, due to the presence of common cold viruses which may lead woman to express higher quantities of HGF into their milk, in order to protect the offspring.

The main strength of this study is that it is one of the largest to date, assessing immune active molecules in colostrum and mature milk. The main limitation of this study is recruitment of women from the general population. As a consequence, very few babies developed eczema. According to UK working party criteria, recurrent rash development reported by the mother is not the most accurate and precise criteria to use. An apparent weakness of this study is that all immunological outcomes were reported by the parents and not based on healthcare professional diagnosis. Mothers tend to over-report health outcomes, such as food allergy in their children, but childhood eczema can be accurately reported by caregivers [44]. The same applies to common cold symptoms as retrospective parental reporting can be inaccurate. Another weakness is that health outcomes were reported by the parents at 6 and 12 months only, with most of the data coming from 6 months. We also did not collect HM on a multiple occasion at many time points, which would be required for the assessment of the actual intake of immune active molecules depending on the duration of breastfeeding.

Our data suggests that another factor substantially increasing the risk of eczematous rash development is the gender of the baby, with girls at higher risk. This result is in agreement with the International Study of Asthma and Allergies in Childhood (ISAAC) phase three data [45], although usually prevalence of eczema during the first year of life varies slightly when compared with older age groups. Differences in perception of eczema by women living in different countries and participating in our study could also play some role in reporting. Some of the previous studies have shown that gender long term does not seem to play a significant role in risk of developing atopy, eczema [42,46] and allergic rhino-conjunctivitis.

## 5. Conclusions

In this large international cohort study of HM immune composition, we found an important association between HM constituents and infant health outcome. Our hypothesis is at least in part confirmed but will require more detailed evaluation of environments. We have also confirmed that variations in levels peptide regulatory factors in human milk do associate with different infant health outcomes. As we focused on an un-selected general population cohort, the power to detect effects on allergy outcomes was limited. Despite this, higher TGFβ and IL13 did associate with eczema. This will require confirmation in a high-risk cohort and could indicate targets for trial of interventions to prevent atopic diseases. 

Future research assessing human milk composition association with immunological health outcomes should include international collaborative studies with strict harmonisation of sampling, storage and analysis protocols between the sites. There is a need in studies, for evaluating human milk immune composition with samples collected at multiple time points prospectively. The only way forward is to bring different research groups together in an attempt to map human milk composition, highlighting differences between the countries and finding associations between human milk immunology and health outcomes.

## Figures and Tables

**Table 1:** Characteristics of study participants and health outcomes between sites of collection.

Characteristics	UK	Russia	Italy	*p*-Value (Three Countries)
Maternal allergic sensitisation *	35/94 (37)	22/156 (14)	9/40 (23)	<0.01 ^a^
Male gender	54/101 (53)	118/216 (55)	41/76 (54)	0.98 ^a^
Primiparous women	55/100 (55)	93/216 (43)	29/75 (39)	0.06 ^a^
Household tobacco smoke exposure	30/99 (30)	135/218 (62)	25/76 (33)	<0.01 ^a^
Parent-reported eczema	20/81 (25)	69/210 (33)	5/47 (11)	<0.01 ^a^
Infant allergic sensitisation *	3/43 (7)	4/156 (3)	7/35 (20)	<0.01 ^a^
Parent-reported food allergy	15/80 (19)	103/210 (49)	3/47 (6)	<0.01 ^a^
Parent-reported common cold	50/101 (50)	117/221 (53)	28/74 (38)	<0.01 ^a^
Parental-reported cough/wheeze	29/101 (37)	43/221 (21)	13/74 (28)	0.02 ^a^

^a^ Pearson χ^2^ test has been used. Data shown are (*n*/(%)) for all binary variables presented; * Defined as skin prick test wheal ≥3 mm to at least one of a panel of common allergens.

**Table 2:** Factors found to be important at least in a single GLM model.

Health Outcome	HM TGFβ2 (ng/mL)	HM HGF (ng/mL)	Detectable HM IL13	Detectable Col IL 13	Sex Baby-Male	Verona	Moscow
Eczema (6 months)	**1.04 (1.01–1.06)**	NI	**0.18 (0.04–0.92)**	NI	**0.2 (0.05–0.84)**	NI	NI
Food allergy (6 months)	NI	NI	NI	**0.10 (0.01–0.83)**	NI	0.13 (0.01–1.46)	2.07 (0.47–9.02)
Cough or wheeze (6 months)	NI	1.89 (0.94–3.78)	NI	NI	NI	NI	NI
Common cold (12 months)	NI	**0.19 (0.04–0.92)**	NI	NI	**4.27 (1.08–16.90)**	NI	**0.02 (0.01–0.23)**

Average importance of the determinants for a particular health outcome during the first year of life, across all possible models. Data for exposures shown to be important presented as OR (95% CI). NI-determinant has not been found to be important for the particular health outcomes development. Statistically significant results presented in bold. GLM = GLmulti; HM = human milk; HGF = Hepatocyte growth factor; TGFβ = transforming growth factor β.

## Appendix A

**Table A1:** Univariate analysis demonstrating demographic data and difference in colostrum and HM cytokine detectability for eczema as a health outcome.

Categorical Variables	No Eczema (*n* = 244)	Eczema (*n* = 94)	*p*-Value
Demographics			
Positive maternal atopy	42 (22.2%)	16 (35.5%)	0.735
Gender (male)	122 (50.8%)	57 (61.3%)	0.111
Multiparity	137 (57.3%)	47 (98.6%)	0.422
Immune active molecules			
Detectable HM IL13	45 (37.8%)	9 (23.8%)	0.002
Detectable Col IL13	52 (24.6%)	16 (36.8%)	0.598
Detectable HM IL2	31 (26.1%)	4 (10.6%)	0.001
Detectable Col IL2	33 (15.6%)	8 (18.4%)	0.355

Pearson χ^2^ test or Fisher’s exact test (if appropriate) were used for this analysis. Statistically significant differences (*p* < 0.05) appear in bold.

**Table A2:** Difference in crude levels of growth factors (ng/mL) for eczema as a health outcome.

Numeric Variables	No Eczema (*n* = 244)	Eczema (*n* = 94)	*p*-Value
HM TGFβ2, median (IQR)	13.57 (14.717)	17.48 (25.619)	0.087
Col TGFβ3, median (IQR)	1.52 (2.513)	2.04 (2.570)	0.169
Col HGF, median (IQR)	2.11 (5.239)	2.52 (6.540)	0.371
HM HGF, median (IQR)	0.79 (0.602)	0.76 (0.745)	0.834
Col TGFβ2, median (IQR)	41.80 (73.124)	47.69 (81.280)	0.663
HM TGFβ3, median (IQR)	0.254 (0.220)	0.302 (0.200)	0.663

Wilcoxon test was used for this analysis. Statistically significant differences (*p* < 0.05) appear in bold.

**Table A3:** Univariate analysis demonstrating demographic data and difference in colostrum and HM cytokine detectability for food allergy as a health outcome.

Categorical Variables	No Food Allergy (*n* = 216)	Food Allergy (*n* = 121)	*p*-Value
Demographics			
Maternal atopy	44 (25.1%)	14 (14.7%)	0.067
Gender (male)	111 (51.9%)	67 (56.8%)	0.457
Multiparity	119 (56.7%)	63 (52.9%)	0.591
Immune active molecules			
Detectable HM IL13	33 (33.7%)	21 (25.9%)	0.337
Detectable Col IL2	46 (25.1%)	22 (21.4%)	0.565
Detectable HM IL2	22 (22.4%)	13 (16.0%)	0.376
Detectable Col IL13	24 (13.0%)	17 (16.5%)	0.530

Pearson χ^2^ test or Fisher’s exact test (if appropriate) were used for this analysis. Statistically significant differences (*p* < 0.05) appear in bold.

**Table A4:** Difference in crude levels of growth factors (ng/mL) for food allergy as a health outcome.

Numeric Variables	No Food Allergy (*n* = 216)	Food Allergy (*n* = 121)	*p*-Value
HM TGFβ2, median (IQR)	13.765 (24.656)	13.616 (12.434)	0.984
Col TGFβ3, median (IQR)	1.718 (2.704)	1.531 (1.854)	0.494
Col HGF, median (IQR)	2.082 (5.436)	2.419 (6.199)	0.437
HM HGF, median (IQR)	0.843 (0.769)	0.731 (0.539)	0.066
Col TGFβ2, median (IQR)	0.289 (0.216)	0.275 (0.207)	0.909
HM_TGFβ3, median (IQR)	0.289 (0.216)	0.275 (0.207)	0.909

Wilcoxon test was used for this analysis. Statistically significant differences (*p* < 0.05) appear in bold.

**Table A5:** Univariate analysis demonstrating demographic data and difference in colostrum and HM cytokine detectability for cough/wheeze as a health outcome.

Categorical Vars	No Cough/Wheeze (*n* = 251)	Cough/Wheeze (*n* = 85)	*p*-Value
Demographics			
Positive maternal atopy	38 (19.7%)	18 (24.0%)	0.541
Gender (male)	128 (51.4%)	48 (58.5%)	0.320
Multiparity	132 (54.3%)	50 (58.8%)	0.554
Immune active molecules			
Detectable HM IL13	40 (29.4%)	14 (33.3%)	0.771
Detectable Col IL2	51 (24.4%)	17 (22.1%)	0.849
Detectable HM IL2	26 (19.1%)	9 (21.4%)	0.825
Detectable Col IL13	31 (14.8%)	10 (13.0%)	0.800

Pearson χ^2^ test or Fisher’s exact test (if appropriate) were used for this analysis. Statistically significant differences (*p* < 0.05) appear in bold.

**Table A6:** Difference in crude levels of growth factors (ng/mL) for cough/wheeze as a health outcome.

Numeric Vars	No Cough/Wheeze (*n* = 251)	Cough/Wheeze (*n* = 85)	*p*-Value
HM TGFβ2, median (IQR)	12.891 (15.595)	21.725 (20.910)	0.090
Col TGFβ3, median (IQR)	1.523 (1.932)	1.890 (3.2648)	0.193
Col HGF, median (IQR)	2.115 (5.413)	2.337 (6.36)	0.564
HM HGF, median (IQR)	0.729 (0.615)	0.859 (0.769)	0.153
Col TGFβ2, median (IQR)	41.274 (64.585)	54.094 (96.060)	0.283
HM_TGFβ3, median (IQR)	0.274 (0.200)	0.314 (0.395)	0.283

Wilcoxon test was used for this analysis. Statistically significant differences (*p* < 0.05) appear in bold.

**Table A7:** Univariate analysis demonstrating demographic data and difference in colostrum and HM cytokine detectability for common cold as a health outcome.

Categorical Vars	No Common Cold (*n* = 143)	Common Cold (*n* = 130)	*p*-Value
Demographics			
Positive maternal atopy	20 (19.0%)	26 (24.1%)	0.469
Gender (male)	76 (54.3%)	69 (53.9%)	1.000
Multiparity	81 (57.9%)	66 (52.0%)	0.399
Immune active molecules			
Detectable HM IL13	28 (31.5%)	13 (21.3%)	0.237
Detectable Col IL2	27 (22.5%)	28 (25.9%)	0.862
Detectable HM IL2	16 (18.0%)	8 (13.1%)	0.501
Detectable Col IL13	20 (16.5%)	16 (14.8%)	0.654

Pearson χ^2^ test or Fisher’s exact test (if appropriate) were used for this analysis. Statistically significant differences (*p* < 0.05) appear in bold.

**Table A8:** Difference in crude levels of growth factors (ng/mL) for common cold as a health outcome.

Numeric Vars	No Common Cold (*n* = 143)	Common Cold (*n* = 130)	*p*-Value
HM TGFβ2, median (IQR)	13.0121 (11.823)	15.67338 (24.983)	0.324
Col TGFβ3, median (IQR)	1.9393452 (2.453)	1.7625169 (4.183)	0.977
Col HGF, median (IQR)	3.4656324 (8.459)	1.997605 (5.249)	0.009
HM HGF, median (IQR)	0.7281423 (0.660)	0.8027212 (0.556)	0.910
Col TGFβ2, median (IQR)	44.1042 (71.380)	42.36296 (77.730)	0.462
HM_TGFβ3, median (IQR)	0.288506 (0.216)	0.2748667 (0.207)	0.909

Wilcoxon test was used for this analysis. Statistically significant differences (*p* < 0.05) appear in bold.
